# The Endoscope, and Its Application to the Diagnosis and Treatment of Urinary Affections

**Published:** 1867-08

**Authors:** A. J. Desormeaux


(*Continued from page 352.*)

several trials, pierced the stricture, located on the bulbous portion of the urethra. There it was pinched and could go no farther. Then, the bougie withdrawn, the patient passed about a cupful of urine, with great difficulty and in scattering drops. The bougie was then reintroduced, left for fifteen minutes, and then urination, like the previous, again took place. Cataplasms were ordered for the lower belly and perineum, and in the evening the patient was somewhat relieved, and passed, by drops, about a half glass of urine; the bougie was reintroduced. He objected to an operation.

*2d Dec.* The same bougie introduced twice during the day; the micturition easier; the bladder sensibly emptied, and occupying only two-thirds of the space between the pubis and umbilicus; sometimes the jet is continuous, but filiform. The urine, however, passes involuntarily in the bed.

*Dec. 3d.* During the night a chill. We left the patient quiet.

*Dec. 10th.* No more chills. The urine is sufficient. There is no complaint of the bladder, and when the desire to urinate occurs, the passage of a few drops gives relief; still dullness on percussion continues over the pubis, there is no fever, the skin is moist and even sweaty. His appetite is gone, a little weak tea (*tisanc*) is his only diet; food is disgusting to him. His lips are swollen, and salivation is abundant. An examination revealed great swelling of the entire mucous membrane, which was covered by a pultaceous layer, surrounded by a red circle, and the parts are very painful.

A portion of chlorate of potash as a gargle, and honey of roses, with chlorhydric acid as a collutorium, are needed.

*Dec. 11th.* By the endoscope, we reached the retracted part of the mucous membrane. It was of a mother of pearl white. In its centre there was a small red point, the opening into the coarctation, very free by the passage of a sound.

The following day the dilatation was continued; Nos. 6, 7, and 8 bougies were used. An erysipelatous phlegmon of the right eyelid occurred and was cured. The patient, however, was always broken down in spirits, and suffering; nevertheless, he passed sufficient urine to keep the bladder more or less free.

*1863, Jan.* During this month, the condition of the patient did not improve, on account of the difficulty offered the treatment, much by the coarctation, which would only admit a filiform bougie (say from two to four-fifths of a line), and by the indocility of the patient.

*Feb. 3d.* The elastic bougies were replaced by the finest of Béniqué's sounds, and gradual dilatation permitted the introduction of No. 35.

Twice, during the month of February, the patient was taken with chills, as at the commencement of the attack, and which yielded to quinine.

The general condition of the patient is bad, the feebleness extreme, there is no appetite, the stools are diarrhœic in their character, so much so, that at the end of February the local treatment had to be suspended to prevent a return of the fever.

*March 10th.* The coarctation is so great that bougies of the elastic gum, used early in January, have not been able to penetrate the bladder. In this first effort we have sometimes been compelled to leave the bougie in front of the retraction. The next day, they cleared it, and gradual dilatation was recommenced with the sounds of Béniqué.

*March 17th.* No. 32 has been reached, but then the patient executes his threat, many times repeated, and demands his exit from the hospital.

*March 21st.* He comes to the consultation, and begs to be permitted to reënter the wards. The stricture is so much reproduced, that the most fine bougies will not penetrate.

*April 3d.* Urethrotomy is practised by aid of the endoscope. This operation presents no difficulty, and is not accompanied with any hemmorrhage; an elastic sound, of medium size, is immediately introduced and fixed in the canal. During the day, the patient withdrew the sound, and no further accidents were presented.

On the morrow, dilatation, or, rather, separation of the lips of the urethral wound, is easily continued.

From this time, his strength, appetite, and a better color of the stools, all unexpected, are evident, and, at the same time, the urethra remains sufficiently dilated without daily catheterism.

*April 20th.* The patient wishes to go to Vincennes, the general condition is not changed and is very satisfactory. The local condition may be appreciated by the fact that, at the time of his departure, a sound No. 41 penetrated the bladder without difficulty.

We have already said that strictures once formed are rarely cured without danger of a relapse; a like cure is almost impossible if the period of granular urethritis is once passed. Relapse, in such cases, is slow to occur; the cure generally lasts for some years; and to keep it up, it is only necessary to pass, at long intervals, a large-sized bougie. On the contrary, when the fibrous retraction occurs, a few days, as in the preceding observation, or a few weeks will suffice to reproduce it after dilatation, and each day, thus to speak, the patient should be sounded. Thus, this is not, properly speaking, a disease threatening a relapse, but one that never cures, and condemns the patient to continual treatment. The first condition is supportable, this is not: and, moreover, when the patient willingly submits himself to it, circumstances of some kind, an intercurrent affection, or some other, occur to compel the cessation of treatment, and then the disease assumes the mastery. It thus becomes necessary to find some more efficacious treatment. To this end, rapid dilatation, cauterization, incision, and excision have been proposed. Of all these means, excision alone is retained. In sudden dilatation, two effects may happen, there is either simple dilatation and a relapse comes on, as after slow dilatation; else there is a tearing of the tissue, and in such case the danger is as great, if not greater, than after urethrotomy. You may recollect a patient, brought here some months since. The next day quick dilatation was resorted to by the inventor of an ingenious instrument; a large urinous phlegmon was the result, and also gangrenous eschars. The patient died in two or three days after entering the hospital.

I have but little to say upon the subject of cauterization as a means of radical cure of coarctation, or of reëstablishing the canal by destroying the new tissues, when the obstacle cannot be passed. I will not repeat all of the opprobrium justly assigned it; one will be sufficient. If it destroys the morbid formation, it is only to replace it by another, more retractile. I do not now call to mind one of my colleagues who is a partisan of so irrational a means. Several times have I encountered the victims of this operation, and never have I seen strictures more difficult to treat, and more rebellious. In all of them the cauterization left them worse than when in the primitive state.

I also throw aside excisions, notwithstanding the injurious inventions to which they owe their birth. They are especially addressed to hypothetical lesions, to very thin valves, the existence of which is not proved, and which must be very rare, in any and every case. This has never assumed a scientific status.

Incision remains to be discussed. As you know, there are two methods by which it may be practiced:—In one, all the parts, from the skin to the urethra, are traversed, this is *external urethrotomy;* in the other, the tissues forming the retraction are cut by instruments introduced into the canal, this is *internal urethrotomy.*

For the first, there are two processes:—It can be performed by directing a cutting instrument upon a conductor; or else in seeking the canal through the soft parts without a conductor. In the first case, it is necessary that the stricture will admit of the introduction of a catheter, whence dilatation is commenced, so that its passage is made possible; and in such case, internal urethrotomy may be performed in the ordinary manner, and with less danger and pain. True, it has been stated that the cures of external urethrotomy are more durable; others, however, have denied this result, and for my part, in two cases in which I have performed this operation, the relapse was not long in taking place; so to speak, it occurred at the same time as the healing of the perineal wound. In a third case, operated upon by one of my colleagues, I was forced to perform the operation of internal urethrotomy for the purpose of removing a coarctation produced immediately after an external urethrotomy had been made. These three examples have been sufficient to debar me from performing this operation.

But external urethrotomy is an operation sometimes necessary, and one which should not be rejected, for it may happen to be the only resource left when all others have failed; it is, however, dangerous, difficult, and, what is worse, uncertain, as you have seen by my observations, when it was tried by a surgeon, who, despite his ability, could not terminate the operation. I thus am forced to believe that one of the greatest merits of the endoscope will often be to replace external urethrotomy, by a process but slightly dangerous.

Now let us turn our attention to internal urethrotomy. Its advantages are calculated to procure a more prompt and certain cure, to afford less chance of relapse, much less severe, in substituting upon the retracted part, a somewhat retractile tissue, for one thick, hard, inodular, and difficult of retracticity. Thus you see that this method should not be resorted to, except in the last stages of coarctation, for inodular retractions.

The modes of internal urethrotomy are, naturally, divided into two, incision from front to rear, and its reverse; let us add a third, endoscopic urethrotomy, the principles of which are different, and which present many special advantages.

To incise a retraction from behind forwards, it is necessary to pass an instrument sufficiently large, and one which is brought forward after the passage of the blade previously concealed. This cannot be used save in coarctations of a certain size, or where dilatation has been effected to a certain extent, of which the length or extent, and accidents found at the commencement of the cure, are not avoided by this method. It does not serve to render the cure quicker, but only more certain. It has been preferred until the incision from front to rear, made by sure instruments, such as those of MM. Sédillat and Maisaneve, made the operation sure. The last instrument you have seen me employ, and you can judge of its excellencies.

It will be of great service; it can act upon very restricted coarctations, and, consequently, without previous preparation in many cases, thanks to the size of it. It requires a conductor, and in some cases, where the opening cannot be found, a long search may be necessary. Small as may be the passage it requires, that passage requires preparation, and this is the most difficult and the longest treatment of the most decided coarctations. Be the treatment what it may, a conductor must be first passed, and often, so as to prepare the passage. The endoscope alone enables us to decide if other instruments will permit us to diagnose correctly. No other method furnishes a certain means of recognizing the disposition of the obstacle. No other process furnishes any means of surely knowing the situation of the obstacle, and, consequently, of judging upon which side it is best to turn the cutting edge of the instrument, whence it often happens, if the accidental tissue only occupies one side of the canal, the incision is made upon the healthy portion. Now, I think it best to cut, in such cases, the diseased parts; they are less sensitive, less vascular, less disposed to immediate adhesion; on this account, I always attack the thickest part for the purpose of making the incision, and, possibly, owing to a strict adherence to this rule, I may owe my success, never having had a serious accident from twenty cases of endoscopic urethrotomy. Before speaking of the advantages possessed by these means, I will describe to you the way of employing them:—

The instruments (uréthrotomes) which I employ are simple probe-pointed bistouries, with a cutting edge of about four lines, the blade small and thin (délié), and in a handle bent at an angle in the stylet I have already described. Having vision to aid us, it is not necessary to employ the same means required by like instruments to conduct the cutting edge and to protect the healthy parts from their action, as without the endoscopic aid. It is necessary to have urethrotomes of two kinds, one with the cutting edge below the handle, the other with the cutting edge above and opposed to the handle. The button or probe point should be no larger than that on the stylet.

Before incising, the opening must be found by means of a stylet, as previously said; then the stylet being withdrawn, the urethrotome is passed to the previously recognized orifice. Once there, by gentle pressure, not by jerks and thrusts, the instrument readily and easily passes. If the passage is tortuous, its cutting edge acting on the more salient points, it makes its own way. Once introduced entirely through the extent of the obstacle, we withdraw it, pressing slightly so that the blade may completely divide the inodular tissue; this done, no more pressure must be used.

If it becomes necessary to reintroduce the instrument, to make a new incision, or to enlarge the previous one, press it towards it back, so that its probe point, in passing the wound, cannot pass from the bounds of the urethra.

Formerly, I made two, and sometimes four incisions; recognizing the futility of this, I now never make but one. When I see two inodular masses at the end of the instrument, I may deem it necessary to divide both.

When we see masses or fibrous filaments at any one point, towards this point the cutting edge must be turned, and we should endeavor to completely divide the pathological structure. If nothing indicates the side to be cut, I choose the upper part of the canal, for, upon this surface, there are less dangers, as Reybard remarks, and, besides, should it become necessary to again operate, there is less chance of the urethrotome passing through the wound, and less danger if it does.

The incision thus made is not very painful; it is slightly, if at all so, when the tissue is inodular. It is also a fact, that in such cases little or no blood is lost, and what is may be promptly arrested.

The only accident I have yet seen, as a consequence of urethrotomy, is an access of intermitting fever, and this I have never found, save in such cases where it had been induced by previous catheterism; so, when compelled to operate upon a patient previously thus affected, I order to be given in the morning, 11½ grains (*60 centigrammes*) of the sulphate of quinine, and 10 drops of laudanum in an infusion of valerian, all by injection. By this treatment, but one accident has supervened, and this though nothing else has been done to prevent a recurrence. There is a curious fact connected with this urethral fever. At certain times, almost all patients sounded are attacked; at others, scarcely any are affected; so it seems to be under epidemic influence, and is genarally most common when intermittents otherwise prevail. In our wards last year, for several months, intermittent affections prevailed, they attacked, indifferently, patients affected in all sorts of ways, fractures, contusions, white-swelling (*tumour blanch*), ophthalmia, etc. The form assumed was sometimes neuralgic, then febrile accesses of fever, generally of the quotidian type, rarely tertian. Whilst this epidemic lasted, the urethral fever affected almost all who had a retention of urine; after urethrotomy, after simple catheterism, after any violent effort to expel the urine, an access would occur. I could, however, continue the treatment, aided by the effect of the quinine, the action of which seemed to be as powerful in such cases as in marsh fever.

As to the care given after an operation, I introduce, immediately after the incision, a large-sized sound, not sufficiently to distend the cut violently, but large enough to keep the incision open, and also to give issue to the urine. I am well aware that some surgeons object to a permanent sound in the urethra, and accuse it of many accidents. So far as I am concerned, I have not observed them. I, moreover, think too much importance has been given to such cases; the resting sound, in such cases, does not seem to me to exert so much influence as is generally attributed to it; it neither possesses the merit some ascribe to it, nor the dangers expected by others; suffice it to say it is painful and its presence inconveniences the sick person, I do not, therefore employ it.

I generally withdraw the sound on the fourth or fifth day; then I complete the cure by the metallic bougies of Béniqué. These bougies are not well adapted to general dilatation, but they seem to act especially upon the affected point.

Urethrotomy, effected by means of the endoscope, partakes of the advantages of the other processes, it has also its peculiar advantages. Like the other treatments, it may cause a radical cure, but more time is necessary after dilatation. There is reason why I should insist upon urethrotomy, a single case that I will shortly report, will fully exemplify that, in certain cases, dilatation affords but temporary relief, whilst incision alone produces a cure.

Endoscopic urethrotomy is preferable to all other means in this, it shortens the treatment, as it does not necessitate any treatment of a preliminary kind to pass the coarctation, and in itself furnishes the means to go through the obstacle, while the methods aiming to act from rear to front require, first, the introduction of a conductor; at the same time, this method enables us to see exactly the position of the stricture, and, of necessity, to properly direct the cutting blade. We have already seen that the endoscope often enables us to decide if urethrotomy is necessary, and to be certain of the fact.

Really, the greatest advantage of this method is, that it furnishes a means to overcome an obstacle, rebellious to all other means. I am convinced, and think you are, that the endoscope will often enable us to discover coarctations, and to practice immediate urethrotomy, in cases where other means would not only require time, but might fail entirely. The endoscope may render unnecessary, or, at least, the necessity very rare, for the puncture of the bladder and external urethrotomy withont a conductor.

The following cases, in addition to those due to M. Civiale, will suffice, I think, to demonstrate the advantages of the new method. It is necessary to state that, in several of these cases, the stricture could not be passed by ordinary means, and, consequently, intra-urethral incision could only be used by means of the endoscope.

*Stricture of the Urethra.—Catheterism, by Ordinary Means, Impossible.—Endoscopic Urethrotomy.—Cure.*

On the 22d of February, 1863, Charles F., 34 years of age, a mechanician, married, came to Necker Hospital.

This man was of a vigorous constitution; as to anterior affections of the urinary passages, he only admits of a slight blennorrhagia, contracted fourteen years previously, and cured in a few days without sequences.

The actual disease was caused by a traumatic effect. In

February, 1862, the man stumbled and fell from half a yard (*demi metre*) in height, upon a bar of iron, striking heavily upon his perineum. Spite of this, he continued his work, but soon the pain became so great that he was compelled to go home. There was weight in the perineum and anxiety to urinate, an anxiety that could not be obeyed. A physician was called, introduced a catheter, and brought off clots of blood. For some days, the sound was introduced from time to time, then its use was suspended, the patient urinating with facility.

The cure seemed complete, there only remained slight pain near the bulb; but in four or five months micturition became painful; the stream diminished daily; and in September, the water passed only by drops.

M. Chaudemont was consulted. He tried, with failure, to introduce bougies, and ultimately recommended external urethrotomy, a process rejected by the patient. Soon the patient, ever tormented with dysuria, went again to M. Chaudemont. Catheterism could not be performed then. After a month of fruitless efforts, M. Chaudemont sent him to me. I several times attempted to introduce the bougie, with like success, or the want of it.

In the first and second explorations of the endoscope, I found the orifice in the bulbous portion, and, on consultation, M. Chaudemont and myself concluded, if another examination was not more happy, an external urethrotomy made by this able surgeon, without a conductor would be necessary, an operation to be practised by him.

So, on the 28th February, a third exploration exhibited, in an irregular surface, mammelonoid of the dead white of coarctation, with restricted orifice at the left inferior parț of the urethra. The orifice was much contracted, and I substituted for the former treatment the whalebone bougie, which I left in place, withdrawing the endoscope. The following day, a very fine bougie could be introduced in the ordinary way.

On the 5th March, the pain forced a cessation of any attempt at catheterism.

March 8th. Aftet a renewed and fruitless attempt at catheterism, urethrotomy was decided on. By aid of the endoscope, I passed a stylet, introduced an urethrotome, and completely divided the inodular tissue, above and to the right, in its densest part. But few drops were lost. An elastic sound No. 13, was then introduced, to remain some time.

Up to the 13th March, the sound was retained without any accident; then it was withdrawn, and a No. 34 Béniqué bougie introduced. Gradually, the size of the bougie was increased; at the end of eight days, No. 54 (*plus de 8 millimetres de diameter*), say three and one-fifth lines English measure, passed readily. The patient having educated himself to pass the sound and his urine readily passing, left the hospital, recommended, however, to occasionally introduce a bougie of large size.

*Stricture of the Urethra without Previous Dilatation.*

T., aged 60 years, a shoemaker and married, was in the Necker Hospital.

This man had been a soldier, and, naturally, addicted to women and wine. When 21 years of age, he contracted his first blennorrhagia, and entered a hospital at Strasbourg, there he was treated by lotions and injections. Two years after, he had a second. He went into a Montpelier hospital, staid there about a month, and left without being cured. The discharge continued for some four months, abundant, accompanied by long and painful erections; he made no change in his habits, as regards coition and the imbibing of alcoholic drinks; he thought he could cure the disease by these excesses. There was neither chancre nor bubo. He kept up, and does still, this mode of life. He has had clap, he knows not how long, and may even have it now. Whenever he drinks too much, urination is difficult, and a white drop appears at the meatus before the urinary emission; little by little, the difficulty of micturition becomes permanently established. In 1849, he entered the hospital at Brest, where he learned to sound himself, and whenever urination became difficult he introduced the sound himself. After that, micturition was easier, but he was not sure that the bougie entered the bladder.

It is now several years since the stricture has reached its present point, that is to say, the man cannot hold his urine when the desire to pass it seizes him; he cannot stop, once its flow has commenced; there is no pain in this, no great effort, but its duration is more or less long; the jet is very fine, sometimes bifurcated, one portion being projected, ejaculated, whilst the other follows vertically. The ejaculation is always made normally. For three years, his condition has been the same.

Upon his entry into the hospital, I attempted to pass bougies Nos. 6, 5, 4, but ineffectually; they could not clear a first coarctation in the spongy portion, but were arrested under the symphysis. Large wax bougies were introduced up to this point; the patient retained them as long as possible, and from them he experienced relief; he urinated more easily, but since the catheterism experiences almost constant pain in the gland; drops of blood flowed from the meatus on the withdrawal of the bougie. During the day, the urethragia continued, his shirt was blood spotted, and, squeezing the urethra, drops would appear.

*26th Feb.* Every day a large bougie was introduced and moved back and forth (à frottement) in the spongy portion. This day, an endoscopic examination was made. The tube passed up to the second retraction, demonstrated a very contracted condition, so much so that the stylet nor a whalebone bougie could pass, save by the point into the orifice; the bougie was left in place; a considerable discharge of blood occurred upon the withdrawal of the instrument; the blood must have come from the first stricture, for the second was seen to be fibrous and not vascular; no blood was seen to come from it. All day the blood flowed drop by drop.

Injections were ordered, say 17.434 Troy grains (5 grammes) of the perchloride of iron in 3086.800 parts of water (200 grammes). In the evening he complained of chilliness, turkey flesh feeling (chair de poule), and headache.

During the month of March he was left quiet, the injections were continued on account of a slight muco-sanguineous discharge, which persisted and was increased by each exploration.

*3d April.* This day, the patient being in good condition, the stricture was cut. The tube was put in place, a bistoury with a superior cutting edge was introduced, and two superior incisions made upward, one to the right the other to the left, the discharge of blood and the pain were but slight. A sound of large size was introduced readily into the bladder and there fixed, with but little pain and no chills. The evening was comfortably passed, with some muco-sanguineous discharge between the borders of the meatus and of the sound.

*5th April.* All goes well, the patient scarcely feels the effects of the operation. The sound is withdrawn; urination is facile.

*April 6th.* He complains of lively pains at the meatus, increased by the passage of urine, slightly difficult at this point. There is some œdema of the bridle and, in separating the lips of the orifice, a small patch of herpetic vesicles. They were cauterized by the nitrate of silver.

*8th April.* The pain is calmed, and there is a renewal of the cauterization.

*9th April.* He urinates well; a bougie of Béniquě, No. 39, is easily introduced into the bladder.

*10th April.* Exit.

*Coarctation of the Bulbous Portion.—Ordinary Catheterism Impossible.—Endoscopic Urethrotomy.*

B. (Désiré), 50 years of age, married, and a house porter.

This man, pale, thin, of a feeble and lymphatic constitution, easily worn out by work and prolonged vigils, had never been guilty of any excess. When 31 years of age, a few days after sexual intercourse, a tumor, the size of a pullet's egg, formed in his right groin. It was red and painful, did not suppurate, and continued for two weeks without preventing work, and that too without either chancre or discharge. He continued to lead an irregular life. Four years since, for the first and last time, he experienced a slight blennorrhagic discharge, a little painful, and one curing itself in from fifteen to twenty days, and that without treatment.

He found himself well until three or four months since, when he perceived his urinary jet slow and very short. Little by little, he was obliged, during the night, to discharge his urine every two hours, with but little at a time, and that voided with difficulty. On the 31st December, after a slight excess, that is, a cup of coffee and a half bottle of wine, urinating became impossible; he also experienced much suffering through the length of the penis and in the perineum, and was forced to keep abed. The pain increased with the mictatory efforts.

When he entered the hospital, 8th January, 1862, the patient suffered somewhat; there was a bearing down weight, also lassitude in the perineum; micturition took place either as a thin thread, or else drop by drop.

Catheterism showed a coarctation near the bulbous part. Bougie No. 4 penetrated it and then rested, pinched by its point. In the perineum, a tolerably hard nodosity was felt.

An endoscopic examination showed a stricture, into which the stylet might be introduced, and upon its left wall an ulceration giving issue to some blood. The catheterism gave rise to no accident.

*11th Jan.* A No. 3 bougie will not penetrate, it comes out twisted and bent upon itself.

*16th.* Every attempt to introduce the same bougie is fruitless. With the endoscope, the stylet was introduced, and then the whalebone bougie, which was fixed in the retracted part, the patient to retain it until the desire to urinate was felt.

*17th.* Yesterday, after having withdrawn the bougie, the patient could urinate more readily, in fact, there was a great amelioration; but in the morning the retraction returned, and at the same point. The bougie could, however, enter and remain, pinched at its point. In the evening, the patient said he was able, during the day, to pass it entirely.

*22d.* Endoscopic examination. Introduction of the whalebone bougie, well protected (bougie de balein gardér), for two hours. The patient does not suffer, the urinal jet is more easy, but scattering.

*23d.* The endoscopic sound was introduced up to the retracted point; at this point a bistoury was inserted, and the indurated tissues, in all of their superior surface, divided. The discharge of blood was but little. Immediately after, a No. 20 sound could pass; it was stopped by an obstacle beyond the prostate, but ultimately reached the bladder. The urine freely flowed, the subject was relieved, though, as a result of the operation, he was chilly, and, with a feeling of faintness, returned to his bed. Cataplasms were applied to the lower abdomen and to the perineum. Soon, heat and good feeling sensations reappeared. A conditional injection of sulphate of quinine was prescribed. In the evening, the patient was in good condition, had urinated well, suffered but little, had no chills, and was in a state of gentle moisture. The injection was not given.

*24th.* Same satisfactory condition. The sound remains in place.

*25th.* Doing well in the morning. Going twice, during the day, to the privy, was seized with a violent chill of about two hours duration, and followed by fever, sweating, indisposition, prostration, and headache. Ordered the following enema:—℞. Valerian 2 gram. (say grs. 80$\frac{1}{4}$), quin. sulph. 50 centgram. (grs. 8), camphor 2 gram. (grs. 80$\frac{1}{4}$), Laudanum of Rousseau gtt. 10

*27th* The patient has done well these two days past, suffering but little, with neither chills nor fever. The sound is withdrawn; the discharge is sero-purulent.

*28th.* The patient urinates easily, and, according to him, with a larger jet than ever before; he might be considered cured, were it not for a slight muco-purulent discharge, and still some pain during, but especially after, micturition.

*3d Feb.* A bougie of white metal, No. 28, (equal to Carrière's 21,) was passed into the bladder.

*4th.* Bougie 29

*13th.* The same bougie introduced each morning, and retained for an instant. An endoscopic examination reveals no cicatrices from the incisions. The condition, general and local, is excellent.

*14th.* Discharged.

*Stricture of the Spongy Portion of the Urethra.*

P. (John), aged 42 years, a shoemaker by trade, married, and in the Necker Hospital.

This man is of good constitution, and has never been sick. At the age of 21, he had an intense blennorrhagia, which appeared after nine days' incubation. He was accustomed to drink largely of beer and wine, without, however, committing many excesses, and exercised himself as a baker, which fatigued him much. He did not change his habits and used no treatment but tisanes; the discharge was abundant, the pains severe, the nights tormented by prolonged erections. Three weeks after, not yet cured, he had new intercourse with women, and renewed them oftener twice than once a week. He also opened a wine store, which did not tend to prevent his drinking. One year after its commencement, the discharge was still abundant. During the second year, it diminished little by little, and existed only in the form of a drop of pus, which he forced out in the morning. This, too, soon left of itself, though he gave up neither wine nor women. He then passed fifteen years without inconvenience. In 1857, he perceived, for the first time, after a slight debauch, that it was difficult for him to urinate, the stream was small, and only voided by great effort. The following days the micturition was more facile, but after every excess, say on an average each eight days, there was a new retention. He consulted a physician, who, fruitlessly, attempted to pass bougies into the bladder, and had caused but little relief; the dysuria increased with each and every excess. The ejaculation was with difficulty made, and meantime the sperm flowed like saliva. During the past two months, these last symptoms are especially manifest. The retention of urine is more complete and accompanied by acute pain. The urine only passes in a fine jet, and that falling perpendicularly, and sometimes in a spiral formation. His general health becoming affected, he concluded, by the advice of his physician, to enter the hospital.

*4th Jan.* A large sound was introduced into the urethra, it passed along the spongy portion, but was checked at the junction of the bulbous and membranous parts; it caused to project

